# Symptomatic Carotid Atheroma Inflammation Lumen-stenosis score compared with Oxford and Essen risk scores to predict recurrent stroke in symptomatic carotid stenosis

**DOI:** 10.1177/23969873231186911

**Published:** 2023-07-22

**Authors:** Sarah Gorey, John J McCabe, Pol Camps-Renom, Nicola Giannotti, Jonathan P McNulty, Mary Barry, Tim Cassidy, Simon Cronin, Eamon Dolan, Alejandro Fernández-León, Shane Foley, Joseph Harbison, Martin O’Connell, David J Williams, Michael Marnane, Joan Martí-Fabregas, Peter J Kelly

**Affiliations:** 1 Health Research Board (HRB), Stroke Clinical Trials Network Ireland (SCTNI), Dublin, Ireland; 2 School of Medicine, University College Dublin, Dublin, Ireland; 3 Stroke Service, Mater Misericordiae University Hospital, Dublin, Ireland; 4 Department of Neurology, Hospital de la Santa Creu i Sant Pau, Biomedical Research Institute Sant Pau (IIB Sant Pau), Universitat Autònoma de Barcelona (Department of Medicine), Barcelona, Spain; 5 Discipline of Medical Imaging Science, Faculty of Medicine and Health, University of Sydney, Camperdown, NSW, Australia; 6 Department of Vascular Surgery, St Vincent’s University Hospital, Dublin, Ireland; 7 Department of Geriatric Medicine, St Vincent’s University Hospital, Dublin, Ireland; 8 Department of Neurology, Cork University Hospital, Cork, Ireland; 9 Department of Clinical Neuroscience, College of Medicine and Health, University College Cork, Cork, Ireland; 10 Department of Geriatric Medicine, James Connolly Memorial Hospital, Dublin, Ireland; 11 Department of Nuclear Medicine, Hospital de la Santa Creu i Sant Pau, Barcelona, Spain; 12 Stroke Service, Department of Geriatric Medicine, St James’ Hospital, Dublin, Ireland; 13 Department of Radiology, Mater Misericordiae University Hospital, Dublin, Ireland; 14 Department of Geriatric and Stroke Medicine, Royal College of Surgeons in Ireland (RCSI), University of Medicine and Health Sciences, Dublin, Ireland

**Keywords:** Prediction, inflammation, recurrent stroke, atherosclerosis, carotid stenosis, vascular inflammation

## Abstract

**Background:**

The Oxford Carotid Stenosis tool (OCST) and Essen Stroke Risk Score (ESRS) are validated to predict recurrent stroke in patients with and without carotid stenosis. The Symptomatic Carotid Atheroma Inflammation Lumen stenosis (SCAIL) score combines stenosis and plaque inflammation on fluorodeoxyglucose positron-emission tomography (^18^FDG-PET). We compared SCAIL with OCST and ESRS to predict ipsilateral stroke recurrence in symptomatic carotid stenosis.

**Patients and methods:**

We pooled three prospective cohort studies of patients with recent (<30 days) non-severe ischaemic stroke/TIA and internal carotid artery stenosis (>50%). All patients had carotid ^18^FDG-PET/CT angiography and late follow-up, with censoring at carotid revascularisation.

**Results:**

Of 212 included patients, 16 post-PET ipsilateral recurrent strokes occurred in 343 patient-years follow-up (median 42 days (IQR 13–815)).

Baseline SCAIL predicted recurrent stroke (unadjusted hazard ratio [HR] 1.96, CI 1.20–3.22, *p* = 0.007, adjusted HR 2.37, CI 1.31–4.29, *p* = 0.004). The HR for OCST was 0.996 (CI 0.987–1.006, *p* = 0.49) and for ESRS was 1.26 (CI 0.87–1.82, *p* = 0.23) (all per 1-point score increase). C-statistics were: SCAIL 0.66 (CI 0.51–0.80), OCST 0.52 (CI 0.40–0.64), ESRS 0.61 (CI 0.48–0.74). Compared with ESRS, addition of plaque inflammation (SUV_max_) to ESRS improved risk prediction when analysed continuously (HR 1.51, CI 1.05–2.16, *p* = 0.03) and categorically (*p_trend_* = 0.005 for risk increase across groups; HR 3.31, CI 1.42–7.72, *p* = 0.006; net reclassification improvement 10%). Findings were unchanged by further addition of carotid stenosis.

**Conclusions:**

SCAIL predicted recurrent stroke, had discrimination better than chance, and improved the prognostic utility of ESRS, suggesting that measuring plaque inflammation may improve risk stratification in carotid stenosis.

## Graphical abstract

**Graphical abstract:**
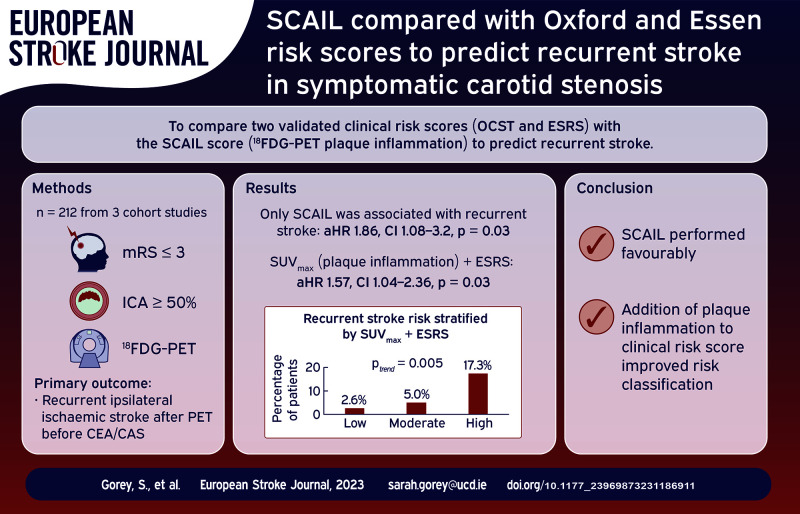

## Introduction

Large artery ischaemic stroke is associated with high-risk of recurrence compared with other stroke subtypes.^1,2^ After subgroup analysis suggested less benefit in some patients, clinical prediction rules were developed to select the most appropriate patients for carotid endarterectomy.^3^ The Oxford Carotid Stenosis tool (OCST) estimates the 5-year risk of recurrent stroke in patients with carotid stenosis without carotid endarterectomy.^4^ Although not specific to carotid stenosis, the Essen Stroke Risk Score (ESRS) has been externally-validated to predict risk of vascular recurrent events in patients with large-artery stroke and other stroke mechanistic subtypes.^5,6^

Accumulating evidence indicates that inflammation is an important contributor to recurrent stroke.^7^ The recently-described Symptomatic Carotid Atheroma Inflammation Lumen stenosis (SCAIL) score combines carotid stenosis severity and quantification of carotid plaque inflammation, derived from measurement of glucose metabolism in metabolically active carotid atheroma using ^18^FDG-PET/CTA.^8,9^ The SCAIL score has been externally-validated to improve identification of patients with early (90-day) and late (5-year) recurrent stroke, compared to stenosis severity alone.^8,10^ However, its prognostic utility compared with established risk scores such as OCST and ESRS is unknown. To further investigate the validity of the SCAIL score, we sought to compare the prognostic utility of the SCAIL score with the ESRS and OCST for recurrent stroke in patients with non-severe stroke/TIA and ipsilateral carotid stenosis. We also investigated whether the addition of plaque inflammation to the clinically-based ESRS and OCST may improve their prognostic utility.

## Patients and methods

### Patients

We pooled individual-participant data from three highly-similar cohort studies: BIOVASC (Biomarkers and Imaging of Vulnerable Atherosclerosis in Symptomatic Carotid Artery Disease), DUCASS (Dublin Carotid Atherosclerosis Stroke Study) and Barcelona Plaque Study. Eligibility criteria were recent (⩽7 days Barcelona, <14 days DUCASS, <30 days BIOVASC) non-severe ischaemic stroke, TIA or retinal artery embolism (mRS⩽3) and ipsilateral internal carotid artery stenosis, identified by duplex ultrasound, CTA and/or MRA. Carotid revascularisation was at the discretion of the treating physician. All participants had ^18^FDG-PET with co-registered CTA performed within 7 days of presentation and prior to revascularisation, according to a standard protocol analysed by a single rater (Web-supplement). All participants were followed-up at 7, 30, 90 days and 1 year, and patients from the DUCASS and BIOVASC cohorts (175 participants, 83% of all) had further late follow-up (average 5 years).

### Outcome

The pre-specified primary outcome was recurrent ipsilateral ischaemic stroke occurring after PET-imaging and before revascularisation (if done) or at the last follow-up assessment, independently adjudicated by an investigator blinded to imaging findings (Web-supplement).

### Assignment of scores

Clinical variables were assigned scores as defined by OSCT and ESRS (Web-supplement). OCST combines the products of 11 clinical items to produce a score, which through use of a normogram (or electronic application) equates to stroke recurrence risk while to generate ESRS the sum of eight clinical risk factors, each worth one point (except 2 for age >75 years) is calculated. OCST includes plaque ulceration assessed by invasive carotid angiography. As stenosis was assessed non-invasively in our study, the primary analysis was performed assuming a randomly assigned 50% of the cohort had ulcerated plaque, with sensitivity analysis assuming prevalence of plaque ulceration of 25%, based on published prevalence data of plaque ulceration in symptomatic carotid stenosis.^11,12^ The SCAIL score was assigned based on degree of carotid stenosis and inflammation measured by the maximum standardised uptake value (SUV_max_) on ^18^FDG-PET in the symptomatic carotid plaque (Table 1).

**Table 1.** SCAIL score.

SCAIL score	Measure	SCAIL points
Plaque SUV_max_, g/ml	<2	0
	2–2.99	1
	3–3.99	2
	⩾4	3
Lumen stenosis,%	<50	0
	50–69	1
	⩾70	2
Sum of scores, maximum possible		5

A SCAIL score of ⩾3 has reported sensitivity of 88.2% and specificity of 45.1% for prediction of recurrent stroke.^10^

Addition of plaque inflammation and both SCAIL variables to clinical scores:

To assess the incremental improvement in prognostic value to the ESRS and OCST by addition of plaque inflammation, the SUV_max_ score items of SCAIL were combined with each score.

We then examined the prognostic utility of the combined ESRS-inflammation score in a categorical fashion, by dichotomising ESRS into high (⩾3) and low (<3), and the SUV_max_ score item dichotomised as high (⩾3) and low (<3) categories based on previously-reported risk thresholds.^10,13^ These were combined to create three new risk categories: Low (low ESRS + low SUV_max_), Moderate (high ESRS + low SUV_max_ *or* low ESRS + high SUV_max_) and High (high ESRS + high SUV_max_).

To further assess prognostic utility, we then added the SCAIL carotid stenosis item to the ESRS only (as OCST already includes an item based on degree of carotid stenosis).

Finally, to assess the incremental prognostic improvement with both addition of plaque inflammation and lumen stenosis to clinical risk scores, we dichotomised the SCAIL score into high and low categories, and combined these with ESRS risk categories. This yielded three new ESRS/SCAIL risk categories, defined as low-risk (low SCAIL < 3 + low ESRS <3), moderate-risk (low SCAIL < 3 + high ESRS⩾3 *or* high SCAIL ⩾ 3+low ESRS < 3) and high-risk (high SCAIL ⩾ 3+high ESRS ⩾ 3). (Web supplement)

### Statistical analysis

The association of each score with first recurrent stroke was analysed using Cox regression analysis, censoring on the date of carotid revascularisation. The proportional hazards assumption was assessed through visual inspection of Kaplan Meier curves (Web Supplement). Unadjusted analysis was performed, followed by adjustment for statin and antiplatelet therapy and then vascular risk factors (age, sex, hypertension, diabetes, smoking). As OCST and ESRS include these variables, we adjusted for statin and antiplatelet therapy only for these scores. Receiver-operating-curve analysis was performed for each score and c-statistics compared using the Chi-squared test. Net reclassification improvement (NRI) from ESRS to the combined ESRS + SUV_max_ score was assessed by cross tabulation and calculated using the equation: $\left(\frac{Events\;upgraded-Events\;downgraded}{All\;Events}\right)+\left(\frac{Non\;Events\;downgraded-Non\;Events\;upgraded}{All\;Non\;Events}\right)$ based on the stratification of risk by each score and the subsequent occurrence or non-occurrence of recurrent stroke outcome events (i.e. appropriate or inappropriate risk reclassification up or down stroke risk categories). Because of methodological limitations about the validity of hypothesis tests for comparison of risk reclassification by prognostic models, no p values are reported for NRI analyses.^14^ A similar approach was taken for the analysis of both SCAIL items (SUV_max_ and stenosis severity) to the ESRS. All analysis was performed using Stata^®^ version 17 (StataCorp LLC, USA).^15^

## Results

### Clinical characteristics

We included 212 participants, 96 BIOVASC (45%), 51 DUCASS (24%) and 65 Barcelona (30%). Carotid stenosis was mild in 21.2% (45), moderate in 43.4% (92), and severe in 35.4% (75). 108 (51%) had carotid revascularisation. After censoring at revascularisation, and excluding early recurrent strokes occurring before PET, there were 16 recurrent ipsilateral strokes in 343 patient-years follow-up. Median follow-up was 42 days (IQR 13–815 days, range 1–3626 days) (Table 2)

**Table 2.** Clinical characteristics.

	Overall *n* = 212	BIOVASC *n* = 96	DUCASS *n* = 51	Barcelona *n* = 65	*p* ^a^
Age	71.3 (SD 9.6)	69.1 (SD 9.5)	71.2 (SD 8.8)	74.6 (SD 9.8)	0.002
Male	154 (72.6%)	67 (69.7%)	38 (74.5%)	49 (75.4%)	0.7
Hypertension	180 (84%)	83 (86.5%)	46 (90%)	51 (78.5%)	0.18
Diabetes mellitus	56 (26.4%)	14 (14.6%)	12 (23.5%)	30 (46.2%)	<0.001
Current smoking	70 (33%)	36 (37.5%)	17 (33.33%)	17 (26.1%)	0.32
Coronary artery disease	44 (20%)	23 (24%)	3 (6%)	18 (27.7%)	0.009
Antiplatelet at study recruitment^b^	174 (82.1%)	90 (93.8%)	35 (68.6%)	49 (75.4%)	<0.001
Statin at study recruitment^b^	170 (80%)	93 (97%)	39 (76.5%)	38 (58.5%)	<0.001
*Clinical event*					<0.001
Retinal artery embolism	12 (6%)	10 (10.4%)	2 (3.9%)	0 (0%)	
Transient ischaemic attack	94 (44%)	50 (52%)	25 (49%)	19 (29.2%)	
Minor stroke (NIH < 5)	91 (43%)	34 (35.4%)	22 (43.1%)	35 (54.8%)	
Major stroke (NIH ⩾ 5)	15 (7%)	2 (2.1%)	2 (3.9%)	11 (16.9%)	
*Carotid Artery stenosis*					<0.001
<*50%*	45 (21.2%)	9 (9.4%)	7 (13.7%)	29 (44.6%)	
*50-69%*	92 (43.4%)	42 (43.8%)	35 (68.6%)	15 (23.1%)	
*⩾70%*	68 (32.1%)	45 (46.9%)	9 (17.7%)	14 (21.5%)	
*Near occlusion*	7 (3.3%)	0 (0%)	0 (0%)	7 (10.8%)	
SUV_max_, Median (IQR)	2.74 (2.3–3.2)	2.84 (2.5–3.3)	2.81 (2.3–3.2)	2.61 (2.3–3.1)	0.11
SCAIL, Median (IQR)	3 (2–3)	3 (2–3)	3 (2–3)	2 (1–3)	0.002
OCST, Median (IQR)	15.5 (8.8–31)	16.2 (9.3–30.3)	16.9 (9.7–45.3)	12.4 (7.3–31.4)	0.28
ESRS, Median (IQR)	3 (2–4)	3 (2–4)	3 (2–4)	3 (3–4)	0.02

a *p* = ANOVA for comparison means; Chi^2^ for comparison of proportions; Kruskal-Wallis for comparison of medians.

b At study recruitment = reflects new antiplatelet and statin therapies started in response to the index stroke/TIA event.

### Comparison of SCAIL, OCST and ESRS

On unadjusted analysis, only SCAIL was associated with recurrent stroke (SCAIL HR 1.96, CI 1.2–3.22, *p* = 0.007; OCST HR 0.996, CI 0.987–1.006, *p* = 0.49; ESRS HR 1.26, CI 0.87–1.82, *p* = 0.23) (all HRs per 1-point increase in score). After adjustment for statin and antithrombotic treatment, the associations were unchanged: SCAIL (HR 1.77, CI 1.08–2.90, *p* = 0.02), OSCT (HR 0.996, CI 0.987–1.006, *p* = 0.53) or ESRS (HR 1.29, CI 0.85–1.95, *p* = 0.23). On further adjustment for vascular risk factors, the association for SCAIL was unchanged (HR 1.86, CI 1.08–3.2, *p* = 0.02) (Table 3). The findings were unchanged on sensitivity analysis assuming 25% of participants had ulcerated plaque, or when ESRS was dichotomised into low (<3) and high-risk (⩾3) categories (Web-supplement). The c-statistic for SCAIL was 0.66 (CI 0.51–0.80), for OCST was 0.52 (CI 0.40–0.64) (*p* = 0.03 for comparison with SCAIL) and for ESRS was 0.61 (CI 0.48–0.74) (*p* = 0.2, for comparison with SCAIL). (Web supplement)

**Table 3.** Crude and adjusted HRs for all scores.

	Crude			Adjusted for antiplatelet and statin			Adjusted for age, sex, hypertension, diabetes, smoking, antiplatelet and statin		
	HR	CI	*p*	HR	CI	*p*	HR	CI	*p*
SCAIL	1.96	1.2–3.22	0.007	1.77	1.08–2.9	0.02	1.86	1.08–3.2	0.03
OCST	0.996	0.99–1.01	0.5	0.996	0.99–1.01	0.53			
ESRS	1.26	0.87–1.82	0.23	1.29	0.85–1.96	0.22			

### Addition of plaque inflammation to ESRS and OCST

When SUV_max_ category was added to ESRS, the prognostic utility of the combined ESRS + inflammation score improved recurrent stroke prediction (HR 1.51, CI 1.05–2.16, *p* = 0.03, all HRs per 1-point increase in score). Findings were consistent after adjustment for statin and antiplatelet therapy (HR 1.57, CI 1.04–2.36, *p* = 0.03) (Table 4). By contrast, addition of the SUV_max_ score to OCST did not improve risk prediction (Table 4).

**Table 4.** Crude and Adjusted HRs for ESRS and OCST combined with SUV_max_.

	Unadjusted			Adjusted for antiplatelet and statin treatment		
	HR	CI	*p*	HR	CI	*p*
ESRS + SUV_max_	1.51	1.05–2.16	0.025	1.57	1.04–2.36	0.03
OCST + SUV_max_	0.99	0.99–1.01	0.5	1.0	0.99–1.01	0.54

**Figure 1.**
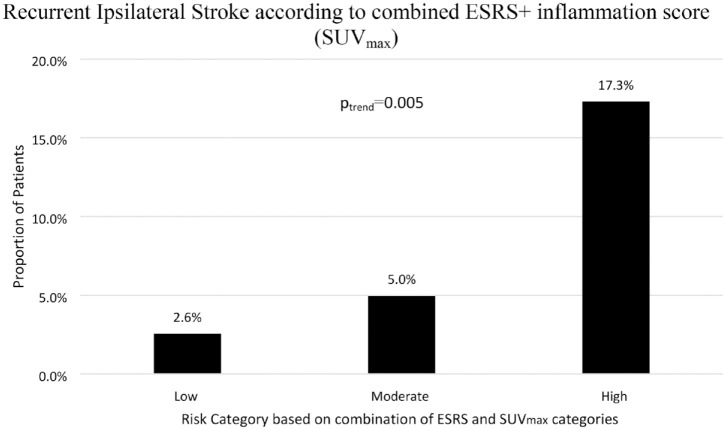
Recurrent Ipsilateral Ischaemic Stroke Risk stratified by combined ESRS + SUV_max_ collapsed into three categories. Risk categories formed by allocating patients according to their ESRS and SUV_max_ scores, defined as: Low: ESRS<3+SUV_max_ <3g/mL; Moderate: ESRS⩾3+SUV_max_<3g/mL *or* ESRS<3 +SUV_max_ ⩾3g/mL; High: ESRS⩾3+SUV_max_ ⩾3g/mL.

When the clinical ESRS score and plaque inflammation (SUV_max_) were dichotomised to create three risk categories (low, ESRS < 3+SUV_max_<3; moderate, ESRS < 3+SUV_max_⩾3 or ESRS ⩾ 3+SUV_max_<3; high, ESRS ⩾ 3+SUV_max_⩾3), the risk of recurrent stroke increased in a step-wise fashion: low, (2.6%, 1/39 patients); moderate, 5% (6/121); high 17.3% (9/52), (*p_trend_* = 0.005) (Figure 1). The HR for recurrent stroke per category increase was 3.31 (CI 1.42–7.72, *p* = 0.006). The high-risk category had sensitivity of 56.3% and specificity of 78.1% to predict recurrent stroke with 76.4% correctly classified (Web supplement). Net reclassification improvement from the ESRS to the new ESRS + inflammation score was 10% (Web supplement). Compared to ESRS alone (c-statistic 0.61, CI 0.48–0.74), the c-statistic for ESRS + SUV_max_ was 0.66 (CI 0.52–0.8) (*p* = 0.2 for comparison).

## Discussion

In the first direct comparison available, only SCAIL identified patients with late recurrent ipsilateral ischaemic stroke with discrimination better than chance compared to OCST and ESRS, indicating that measurement of carotid plaque inflammation using ^18^FDG-PET added important prognostic value. When added to the clinical variables in the validated ESRS score, plaque inflammation (with and without the addition of lumen stenosis) improved risk reclassification, and allowed identification of patients at low, medium and high risk for recurrent stroke. This suggests that measurement of carotid plaque inflammation using PET-CTA potentially provides additional prognostic information to the information provided by clinical risk scores.

Inflammation is important in development and rupture of atherosclerotic plaque.^16^ Building on evidence from observational, genetic, and animal studies, randomised trials have demonstrated that anti-inflammatory therapy with the interleukin-1 antagonist canakinumab, and the broad-spectrum anti-inflammatory agent colchicine reduces vascular events, including stroke, in patients with coronary atherosclerosis.^7,17,18^ The CONVINCE trial is near completion and will report on the efficacy of colchicine to reduce recurrent vascular events after stroke. Other secondary stroke prevention trials of colchicine are beginning.^19^ Consistent with these advances, non-invasive imaging of plaque inflammation may improve risk stratification of patients with carotid atherosclerosis compared with measurement of lumen stenosis or clinical risk scores.

Carotid ^18^FDG-PET is a promising technique to improve selection of patients for randomised trials of carotid revascularisation where benefit is currently uncertain. However, the translation of ^18^FDG-PET scanning to clinical practice is limited by availability and cost, it may be more feasible to use in the research setting. Other imaging techniques, such as identification of intraplaque haemorrhage (another imaging biomarker of plaque inflammation) on high resolution magnetic resonance imaging has been strongly associated with recurrent ipsilateral stroke in patients with symptomatic mild to moderate carotid stenosis.^12^ The combination of these two imaging modalities may yield complementary information and hybrid PET/MRI scanners will facilitate comprehensive atherosclerotic plaque assessment in future.^9^

Strengths of our study include late follow-up in most patients and high rates of contemporary medical therapies, in contrast to older endarterectomy trials. Images were centrally analysed by a single rater to minimise misclassification bias. We acknowledge some limitations. Decisions for carotid revascularisation were not mandated by a central protocol and were at the discretion of treating clinicians, which may have introduced some variability. Additionally, reasons for performing or avoiding carotid revascularisation were not systematically collected, precluding sensitivity analysis. Similarly, carotid plaque ulceration was not routinely measured or reported across study sites. Censoring at revascularisation, while avoiding the bias introduced by peri-procedural stroke events, may have reduced statistical power for some analyses. We acknowledge that the ESRS was not specifically validated in patients with carotid stenosis, although patients with carotid atherosclerosis were included in existing validation studies of ESRS. In our study, the distribution of OSCT scores was towards the lower part of the risk spectrum and patients were treated with high rates of statins and other preventive therapies compared to the original OCST validation cohort, which may have contributed to our OCST findings. The SCAIL score was originally derived and validated in this cohort, so independent replication of our findings in other cohorts is recommended.

The SCAIL score has demonstrated validity to predict early and late recurrent stroke. In our study, SCAIL improved identification of patients with recurrent stroke when directly compared to established clinical risk scores.^8,10^ However, SCAIL correctly classified just over half of patients in our study, and the addition of clinical variables in the ESRS provided only modest further improvements. Prediction models including measures of vascular inflammation, either alone or in combination with clinical risk factors, need to be further refined and validated before they can be applied in clinical practice for patient selection for carotid revascularisation

## Conclusion

SCAIL performed favourably when compared to established clinical risk scores to predict recurrent stroke in patients after minor stroke/TIA and symptomatic carotid stenosis before revascularisation and provided added value for prognosis when added to the clinically-based ESRS. Further studies are needed to determine the role of ^18^FDG-PET to assess plaque inflammation in selection of patients in future randomised trials of carotid revascularisation.

## Supplemental Material

sj-docx-1-eso-10.1177_23969873231186911 – Supplemental material for Symptomatic Carotid Atheroma Inflammation Lumen-stenosis score compared with Oxford and Essen risk scores to predict recurrent stroke in symptomatic carotid stenosis Click here for additional data file. Supplemental material, sj-docx-1-eso-10.1177_23969873231186911 for Symptomatic Carotid Atheroma Inflammation Lumen-stenosis score compared with Oxford and Essen risk scores to predict recurrent stroke in symptomatic carotid stenosis by Sarah Gorey, John J McCabe, Pol Camps-Renom, Nicola Giannotti, Jonathan P McNulty, Mary Barry, Tim Cassidy, Simon Cronin, Eamon Dolan, Alejandro Fernández-León, Shane Foley, Joseph Harbison, Martin O’Connell, David J Williams, Michael Marnane, Joan Martí-Fabregas and Peter J Kelly in European Stroke Journal

sj-docx-2-eso-10.1177_23969873231186911 – Supplemental material for Symptomatic Carotid Atheroma Inflammation Lumen-stenosis score compared with Oxford and Essen risk scores to predict recurrent stroke in symptomatic carotid stenosis Click here for additional data file. Supplemental material, sj-docx-2-eso-10.1177_23969873231186911 for Symptomatic Carotid Atheroma Inflammation Lumen-stenosis score compared with Oxford and Essen risk scores to predict recurrent stroke in symptomatic carotid stenosis by Sarah Gorey, John J McCabe, Pol Camps-Renom, Nicola Giannotti, Jonathan P McNulty, Mary Barry, Tim Cassidy, Simon Cronin, Eamon Dolan, Alejandro Fernández-León, Shane Foley, Joseph Harbison, Martin O’Connell, David J Williams, Michael Marnane, Joan Martí-Fabregas and Peter J Kelly in European Stroke Journal
