# Supplementary material for: Symptomatic Carotid Atheroma Inflammation Lumen-stenosis score compared with Oxford and Essen risk scores to predict recurrent stroke in symptomatic carotid stenosis
Source: Eur Stroke J. 2023 Jul 22;8(4):1064–70. doi: 10.1177/23969873231186911 (PMC10683720; doi:10.1177/23969873231186911)
Supplement: sj-docx-1-eso-10.1177_23969873231186911 – Supplemental material for Symptomatic Carotid Atheroma Inflammation Lumen-stenosis score compared with Oxford and Essen risk scores to predict recurrent stroke in symptomatic carotid stenosis [file sj-docx-1-eso-10.1177_23969873231186911.docx]

STROBE Statement—checklist of items that should be included in reports of observational studies

|  | Item No. | Recommendation | Page  No. | Relevant text from manuscript |
| --- | --- | --- | --- | --- |
| **Title and abstract** | 1 | (*a*) Indicate the study’s design with a commonly used term in the title or the abstract | Abstract, pg 2  Methods pg 4 | “cohort study” |
|  |  | (*b*) Provide in the abstract an informative and balanced summary of what was done and what was found | Pg 2 |  |
| Introduction | | | |  |
| Background/rationale | 2 | Explain the scientific background and rationale for the investigation being reported | Pg 4 | “…to investigate the validity of the SCAIL score we sought to compare the prognostic utility of the SCAIL score with the ESRS and OCST….” |
| Objectives | 3 | State specific objectives, including any prespecified hypotheses | Pg 4 | As above and  “… investigated whether the addition of plaque inflammation to the clinically-based ESRS and OCST may improve their prognostic utility” |
| Methods | | | |  |
| Study design | 4 | Present key elements of study design early in the paper | Pg 4-5 | “pooled 3 highly similar cohort studies” |
| Setting | 5 | Describe the setting, locations, and relevant dates, including periods of recruitment, exposure, follow-up, and data collection | Pg 4-5 | “Patients and methods” section |
| Participants | 6 | (*a*) *Cohort study*—Give the eligibility criteria, and the sources and methods of selection of participants. Describe methods of follow-up  *Case-control study*—Give the eligibility criteria, and the sources and methods of case ascertainment and control selection. Give the rationale for the choice of cases and controls  *Cross-sectional study*—Give the eligibility criteria, and the sources and methods of selection of participants | Pg 5 | 1. Cohort study   “Eligibility criteria were…”  Exposure= “all participants had 18FDG-PET…”  Follow-up= “all participants were followed-up at…” |
|  |  | (*b*) *Cohort study*—For matched studies, give matching criteria and number of exposed and unexposed  *Case-control study*—For matched studies, give matching criteria and the number of controls per case |  | Not a matched study – N/A |
| Variables | 7 | Clearly define all outcomes, exposures, predictors, potential confounders, and effect modifiers. Give diagnostic criteria, if applicable | Pg 5 | Outcome “the pre-specified primary outcome was recurrent ipsilateral ischaemic stroke…”  Exposure: “18FDG-PET with co-registered CTA performed within 7 days of presentation…” |
| Data sources/ measurement | 8* | For each variable of interest, give sources of data and details of methods of assessment (measurement). Describe comparability of assessment methods if there is more than one group | *Pg 5* | *Assignment of scores. (further detail in Web Supplement)* |
| Bias | 9 | Describe any efforts to address potential sources of bias |  | Measurement bias is limited by centralised blinded assessment of CT Angiogram/PET scans  Information bias is limited by standardised and comprehensive colletion of covariates and potential confounders. |
| Study size | 10 | Explain how the study size was arrived at |  | This is a sample size of convenience based on available data from observation study. |

Continued on next page

| Quantitative variables | 11 | Explain how quantitative variables were handled in the analyses. If applicable, describe which groupings were chosen and why | Pg6, | Mean and SD, or median and IQR were used to summarise continuous variables. Proportions of covariates are reported in Table 1. |
| --- | --- | --- | --- | --- |
| Statistical methods | 12 | (*a*) Describe all statistical methods, including those used to control for confounding | Pg 6 |  |
|  |  | (*b*) Describe any methods used to examine subgroups and interactions | Web Supplement |  |
|  |  | (*c*) Explain how missing data were addressed | Pg 6 | Survival analysis |
|  |  | (*d*) *Cohort study*—If applicable, explain how loss to follow-up was addressed  *Case-control study*—If applicable, explain how matching of cases and controls was addressed  *Cross-sectional study*—If applicable, describe analytical methods taking account of sampling strategy |  | d) Cohort study  survival analysis used – time to last follow up varied across patients  Censored at date of revascularisation for those who underwent revascularisation. |
|  |  | (*e*) Describe any sensitivity analyses | Web Supplement | Sensitivity analysis performed to examine effect of different prevalence of carotid plaque ulceration. |
| Results | | | | |
| Participants | 13* | (a) Report numbers of individuals at each stage of study—eg numbers potentially eligible, examined for eligibility, confirmed eligible, included in the study, completing follow-up, and analysed |  | Retrospectively constructed cohort- only patients with PET scan performed from the original cohort studies were included. |
|  |  | (b) Give reasons for non-participation at each stage |  |  |
|  |  | (c) Consider use of a flow diagram |  |  |
| Descriptive data | 14* | (a) Give characteristics of study participants (eg demographic, clinical, social) and information on exposures and potential confounders | Pg 7 and pg XX | “ Results” and  “Table 1” |
|  |  | (b) Indicate number of participants with missing data for each variable of interest |  |  |
|  |  | (c) *Cohort study*—Summarise follow-up time (eg, average and total amount) | Pg 7 | Median follow up was 4 years (IQR 1.2-6.2) but after censoring at revascularisation, this was reduced to 1.6 years (343 patient-years follow up) |
| Outcome data | 15* | *Cohort study*—Report numbers of outcome events or summary measures over time | *Pg 7* | *16 recurrent ipsilateral strokes (primary outcome)* |
|  |  | *Case-control study—*Report numbers in each exposure category, or summary measures of exposure |  |  |
|  |  | *Cross-sectional study—*Report numbers of outcome events or summary measures |  |  |
| Main results | 16 | (*a*) Give unadjusted estimates and, if applicable, confounder-adjusted estimates and their precision (eg, 95% confidence interval). Make clear which confounders were adjusted for and why they were included | Pg 7-8 | Unadjusted and adjusted HR with CI reported. Rationale for adjustment approach in statistical /methods section. |
|  |  | (*b*) Report category boundaries when continuous variables were categorized | Pg 5  Pg 7-8 | Categories (of ESRS and SCAIL) based on previously-reported risk thresholds. |
|  |  | (*c*) If relevant, consider translating estimates of relative risk into absolute risk for a meaningful time period | Pg 8 |  |

Continued on next page

| Other analyses | 17 | Report other analyses done—eg analyses of subgroups and interactions, and sensitivity analyses | Pg 7-8 |  |
| --- | --- | --- | --- | --- |
| Discussion | | | | |
| Key results | 18 | Summarise key results with reference to study objectives | Pg 8 | Paragraph 1 of discussion |
| Limitations | 19 | Discuss limitations of the study, taking into account sources of potential bias or imprecision. Discuss both direction and magnitude of any potential bias | Pg 9 | “We acknowledge some limitations…” |
| Interpretation | 20 | Give a cautious overall interpretation of results considering objectives, limitations, multiplicity of analyses, results from similar studies, and other relevant evidence | Pg 9 |  |
| Generalisability | 21 | Discuss the generalisability (external validity) of the study results | Pg 9 | “comparison of these scores in an independent population would be preferable” |
| Other information | |  | | |
| Funding | 22 | Give the source of funding and the role of the funders for the present study and, if applicable, for the original study on which the present article is based | Pg 10 | Disclosures, funding contributorship, |

*Give information separately for cases and controls in case-control studies and, if applicable, for exposed and unexposed groups in cohort and cross-sectional studies.

**Note:** An Explanation and Elaboration article discusses each checklist item and gives methodological background and published examples of transparent reporting. The STROBE checklist is best used in conjunction with this article (freely available on the Web sites of PLoS Medicine at http://www.plosmedicine.org/, Annals of Internal Medicine at http://www.annals.org/, and Epidemiology at http://www.epidem.com/). Information on the STROBE Initiative is available at www.strobe-statement.org.
