# Supplementary material for: Symptomatic Carotid Atheroma Inflammation Lumen-stenosis score compared with Oxford and Essen risk scores to predict recurrent stroke in symptomatic carotid stenosis
Source: Eur Stroke J. 2023 Jul 22;8(4):1064–70. doi: 10.1177/23969873231186911 (PMC10683720; doi:10.1177/23969873231186911)
Supplement: sj-docx-2-eso-10.1177_23969873231186911 – Supplemental material for Symptomatic Carotid Atheroma Inflammation Lumen-stenosis score compared with Oxford and Essen risk scores to predict recurrent stroke in symptomatic carotid stenosis [file sj-docx-2-eso-10.1177_23969873231186911.docx]

**Web Supplement**

**Additional methods**

**Description of the study cohorts**

The three highly similar cohorts included in this study have been previously described. ^1,2^

BIOVASC study design

BIOVASC was a multicentre prospective cohort study conducted at 10 centres in Ireland (6 sites), Barcelona, Paris, Calgary, and Singapore (1 site each). This analysis includes data from the 6 Irish sites only.

Inclusion criteria were (1) age ≥50 years, (2) presentation to medical attention with recent (<30 days) non severe ischaemic stroke (modified Rankin Scale score ≤3) or motor/speech/vision TIA, (3) ipsilateral carotid artery stenosis (>50% lumen-narrowing) on admission doppler ultrasound, magnetic resonance angiogram (MRA) or computed tomography angiography (CTA) done for clinical care, and (4) PET/CTA completed. The study was approved by participating -hospital Ethics Committees and patient gave informed consent to participate.

All patients had a baseline assessment by trained study personnel and ^18^FDG-PET/CTA <7days of study enrolment. Treating clinicians were advised to provide medical and revascularisation treatments according to guidelines.

DUCASS study design

The DUCASS study was performed in Dublin, Ireland between 2008 and 2011. Eligibility criteria and study methodology for DUCASS were near-identical to BIOVASC, except that imaging was done at a single centre, and the time interval allowed between qualifying stroke/TIA and study entry was 14 days.

Barcelona Plaque Study design

The Barcelona Study of Carotid Plaque Vulnerability (NCT03218527) was an observational study of consecutive adult patients with a recent ischaemic stroke and at least one atherosclerotic plaque in the internal carotid artery on the side consistent with the stroke symptoms, conducted between October 2015 and March 2018. Methods were also near-identical to BIOVASC, except that the interval between qualifying stroke/TIA and study inclusion was 7 days and CTA was usually done before PET/CT at a single centre.

Exclusion criteria

The main exclusion criteria for all three studies were:

1. pregnancy, (2) age <50years, (3) active malignancy, (4) prior neck irradiation or ipsilateral CEA/stenting, (5) ipsilateral carotid occlusion, (6) dementia, (7) renal impairment (eGFR <60ml/min) or other contraindication to contrast-enhanced CT or MRI, (8) other co-morbidities with estimated life-expectancy <1year.

All participants in all three studies were followed-up prospectively for recurrent ischaemic stroke, with follow-up visits at 7, 30, 90 days and 1 year. For this current analysis, 147 of the participants came from Irish studies (BIOVASC and DUCASS) and 5 year follow-up was completed on these patients. 1 year follow-up was completed on the 65 participants included from Barcelona.

**Study definitions in the three cohorts**

Index event: The index event was defined as the first stroke or TIA (motor, speech or unequivocal vision loss) within 30 days of the clinical event (stroke or TIA) immediately preceding presentation to hospital. If a patient reported one or more episodes consistent with TIA or minor stroke in the 30-day period prior to the event immediately preceding medical presentation, the first episode was classified as the Index event, with subsequent new stroke or TIA events classified as Outcome events, only if they occurred after PET scanning had been performed. If no earlier TIA or stroke events had occurred within this period, the event immediately preceding medical presentation was classified as the Index event.

Index Stroke definition: The WHO definition of stroke was used: “rapidly developing clinical signs of focal (at times global) disturbance of cerebral function, lasting more than 24 hours, with no apparent cause other than that of vascular origin”. Ischaemic stroke was confirmed by brain imaging in all cases (CT or MRI).

Index TIA: TIA was defined as acute loss of focal cerebral or ocular function lasting <24 hours presumed, after investigation, due to embolic or thrombotic vascular disease. Where brain imaging (CT or MRI) showed evidence of acute infarction associated with brief focal symptoms, the event was coded as a stroke.

In Table 1 in the main manuscript, index clinical events are characterised as either retinal artery embolism, transient ischaemic attack (TIA) as defined above, or ischaemic stroke (as defined above) classified as minor (NIHSS score <5) or major (NIHSS score ≥5)

Recurrent stroke: Recurrent stroke was defined as a sudden-onset worsened neurological deficit lasting greater than 24 hours occurring after the initial acute stroke or TIA. Gradual progression of an existing neurological deficit, sub-clinical detection of new infarction on neuroimaging, or deterioration due to cerebral oedema, large haemorrhagic transformation, seizure, metabolic disturbance or other illness were excluded.

**Primary outcome**

Primary outcome for this analysis was ipsilateral recurrent ischaemic stroke after PET scanning but before revascularisation (if revascularisation was performed), and in those who did not have revascularisation performed, ipsilateral ischaemic stroke occurring after PET. Web Table 2 shows the primary outcome (ipsilateral recurrent ischaemic stroke, after PET, before revascularisation or if revascularisation not performed, per carotid stenosis category)

OCST was derived and validated to estimate stroke risk to inform selection of patients at high risk without revascularisation. We aimed to compare the SCAIL and other scores for prediction of stroke risk in patients who have not had carotid revascularisation, and therefore we censored at the time of revascularisation, if done. Carotid revascularisation was done in 108 (51%) of participants. Prior to censoring 20 recurrent ischaemic stroke occurred after PET, in 835 patient-years of follow up. After censoring , 16 recurrent ischaemic strokes occurred after PET, in 343 patient-years of follow-up.

Outcome adjudication

All cases of suspected outcomes were confirmed by in-person evaluation by an experienced Stroke Physician. Outcome events were then independently adjudicated by a study investigator (PK in Irish sites and PCR in Barcelona) blinded to imaging data.

**Imaging**

Carotid stenosis ascertainment

Patients with stenosis were identified based on reports of carotid ultrasound, CTA, MRA or digital subtraction angiography reports. Patients were classified as mild, moderate or severe stenosis by reporting radiologists. To verify and standardise measurement of stenosis severity, CTA images were subsequently centrally assessed by a single trained reader (NG). Carotid stenosis was graded using the NASCET approach as mild (<50%), moderate (50-69% lumen narrowing) or severe (70% narrowing or greater) by comparison of the lumen diameter at the maximum site of stenosis to that of distal normal-appearing carotid artery. ^3^

Internal carotid stenosis severity was determined by CT angiography in 137 (64.6%) of participants. In the remaining 75, stenosis was assessed by carotid doppler ultrasound or MR angiography. Details are presented in Web Table 3

Near occlusion

Radiology reports from participating sites were screened for mention of near-occlusion with or without full collapse. Reports using the terms: “near-occlusion”, “string-sign”, “string-like low flow” or similar were coded as near occlusions.

^18^Flurodeoxyglucose positron emission tomography scanning

^18^FDG-PET/CTA was performed according to a standard protocol after a minimum 6-hour fast.^4^ 320MBq of ^18^FDG was administered 2 hours before image acquisition. The uptake time was standardised with the patient resting. PET images were acquired in a 3-dimensional mode in two bed positions for 10 minutes each. After PET, a low-dose CT for attenuation correction was performed using the same scanner followed by an aortic arch to skull-base carotid CTA using contrast bolus tracking. CTA slides (0.625-0.75mm slice thickness, with contrast enhancement) were acquired from the aortic arch to skull base, with reconstruction of axial CTA images at 1mm intervals for analysis.

Standardisation and quality assurance (QA) of PET-CT imaging was achieved across study sites by two strategies: First, participating sites performed internal QA checks of measured compared with actual ^18^F concentrations using standardised ‘phantoms’ which simulate foci of high FDG uptake against background body update to verify adherence to recommended accuracy stantatds.^5^ Second, a pre-specified standardised PET acquisition protocol was used in all study centres, and protocol violations were ineligible for analysis after central imaging review.

Image analysis

Semi-automated co-registration of PET and CTA images was performed, as previously described.^6^ ^18^FDG activity in 10 regions of interest were defined relative to the slice of maximal stenosis. ^18^FDG activity was quantified using standardised uptake values (SUV [g/ml]) =measured uptake [MBq/ml]/injected dose [MBq] per patient weight [g]. We defined the single ‘hottest slice’ (SHS) as the axial slice with the maximal SUV uptake (SUV_max_) and the most diseased segment (MDS) and the single hottest slice plus the adjacent proximal and distal axial slices, corresponding to vessel areas 3mm in length. All images were centrally analysed by a single trained reader (NG). Intra-rater reliability assessment showed excellent agreement (intraclass correlation alpha=0.814, p<0.001). For all analyses relating to plaque inflammation-related metabolism, SUV_max_ was considered as the primary exposure variable of interest.

**Assignment of Scores**

Scores were assigned to data variables as outlined in Web Table 1, with minor modifications described as follows.

Oxford Carotid Stenosis Tool (OCST) : For 20 participants, degree of ipsilateral carotid stenosis was recorded as 50-69% and a score of 2.6 (mid-point of 2.4 and 2.8) was assigned to these participants for this item. Data on presence of ulcerated plaque was not available as routine invasive carotid angiography was not performed in this cohort. Therefore, we randomly assigned 50% of the cohort to a score of 2 the ulcerated plaque item for the primary analysis. Sensitivity analysis was performed repeating the analysis assuming 25%, 75% or 100% of the participants had ulcerated plaque, assigned at random. Results of this sensitivity analysis are presented below.

In the original derivation of OCST, major stroke was defined as stroke with symptoms persisting for at least 7 days. In our study, complete data on duration of symptoms beyond 24 hours was unavailable. We modified this item by coding strokes with NIHSS ≥5 as major, and <5 as minor.

The SCAIL score and its components of carotid lumen stenosis score and carotid inflammation score (SUV_max_) are outlined in Table 4 in the main manuscript.

ESRS has previously been validated in a population of over 15000 to predict recurrent cardiovascular events at 1 year. ^7^ There was a stepwise increase in risk of events with increasing ESRS score, from 2.4% event rate with a score of 0, 5.3% event rate at a score of 3 and 9.2% event rate at a score of 6**.** The original derivation of ESRS suggested dichotomising the score at <3 (corresponding to <4% recurrent stroke risk per annum) or ≥3 (≥4% recurrent stroke risk per annum). ^8^

**Supplementary Results:**

**OCST distribution:**

The mean OCST in this cohort was 44.33 with a range of 3 to 347. However the distribution of OCST in this cohort was non-normally skewed to the “low-risk” end of the scale, thus median and IQR were used as summary statistics in Web Table 1. The median OCST (assuming 50% ulcerated plaque) in this cohort was 15.5 (IQR 8.8 – 31, range 3 – 347)

**Sensitivity analysis for OCST**

As invasive carotid angiography was not routinely performed, sensitivity analysis assuming 25% prevalence of carotid plaque ulceration was performed for OCST. The results are presented in Web Table 6.

**Sensitivity analysis of ESRS by high-risk versus low-risk category**

ESRS was also dichotomised into high risk (>3) and low-risk (<3) categories^8^. 63 (29.7%) were categorised as low-risk with a score of <3 while 149 (70.3%) were in the high-risk category with a score ≥3. Cox regression was repeated with ESRS as a binary variable. Results are presented in Web Table 7.

Comparison of area under the receiver operating curve and c-statistics for the three scores SCAIL, ESRS and OSCT is presented in Web Figure 2.

**Addition of both SCAIL items to the ESRS:**

When ESRS was combined with both stenosis and inflammation severity (i.e. both SCAIL items), the prognostic utility was also improved (HR 1.6, CI 1.14-2.25, p=0.007). This finding remained after adjustment for antiplatelet and statin treatment (HR 1.55, CI 1.09-2.21, p=0.015). (Web Table 8)

When the combined ESRS+SCAIL score was analysed categorically, by dichotomising ESRS and SCAIL to create three new risk groups, (low, ESRS<3+SCAIL<3; moderate, ESRS<3+SCAIL≥3 *or* ESRS≥3+SCAIL<3; high, ESRS≥3+SCAIL≥3) the HR for recurrent ipsilateral stroke per increase in category was 4.22 (CI 1.65-10.82, p=0.003). The risk of recurrent stroke again increased in a stepwise fashion: low, 3.4% (1/29 patients); moderate, 3.6% (4/112); high, 15.5% (11/71) (p_trend_=0.005). (Web Figure 3) Net reclassification improvement for the movement of patients to the combined ESRS+SCAIL score categories from ESRS was 9%.(Web Figure 4)

Compared to ESRS alone (c-statistic 0.61), the c-statistic for ESRS+SCAIL was 0.69 (CI 0.55-0.82).

The sensitivity and specificity of each score and combinations of scores to accurately predict recurrent ischaemic stroke are presented in Web Table 5. Comparison of c-statistics is presented in Web Table 10

.

**Tables and Figures**

Web Table 1 OCST and ESRS scores

Web Table 2 Primary outcome by degree of stenosis and relative to revascularisation decision

Web Table 3 Carotid stenosis and method of detection

Web Table 4 Modality of carotid imaging and revascularisation

Web Table 5 Baseline clinical characteristics of cohort by outcome or no outcome

Web Table 6 Sensitivity analysis OCST, assuming prevalence 25% plaque ulceration

Web Table 7 HR for ESRS, dichotomised into low and high risk groups

Web table 8 Association of combinations of ESRS with stenosis alone and both SCAIL items, with recurrent ipsilateral ischaemic stroke

Web table 9 Sensitivity and Specificity of Each Score and Combinations of Scores

Web Table 10 Comparison of Discrimination of each score and combination by comparison of C-statistics

Web Figure 1 Kaplan Meier curves for a) OCST (dichotomised at median), (b) ESRS <3 and ≥3, (c) SCAIL <3 and ≥3.

Web Figure 2 Area under the receiver operating curve for comparison of c-statistics for SCAIL, OCST and ESRS

Web Figure 3 Recurrent Ipsilateral Stroke rate per category created by combination of ESRS and SCAIL

Web Figure 4 Net Reclassification Matrix

| **Web Table 1 (a) Oxford Carotid Stenosis Tool** | | **(b) Essen Stroke Risk Score** | |
| --- | --- | --- | --- |
| **Stenosis** (per 10% )  50-59%  60-69%  70-79%  80-89%  90-99%  **Near Occlusion**  **Irregular or ulcerated carotid plaque**  **Male**  **Age** (per 10 years)  31-40  41-50  51-60  61-70  71-80  81-90  **Time Since last event**  0-13 days  14-28 days  29-89 days  90-365 days  **Presenting event**  Ocular  Single TIA  Multiple TIA  Minor Stroke  Major Stroke*  **Hypertension**  **Diabetes mellitus**  **PVD**  **Previous myocardial infarction** | 2.4  2.8  3.3  3.9  4.6  0.5  2  1.2  1.1  1.2  1.3  1.5  1.6  1.8  8.7  8  6.3  2.3  1  1.4  2  1.8  2.5  1.2  1.4  1.2  1.6 | **Age**  <65 years  65-75 years  >75 years  **Additional Stroke or TIA**  **Smoker** (yes)  **Hypertension**  **Diabetes mellitus**  **Any cardiovascular disease** (except MI or AF)  **Peripheral artery disease**  **Previous myocardial infarction** | 0  1  2  1  1  1  1  1  1  1 |
| **Product of scores, maximum possible** | 348.5 | **Sum of scores, maximum possible** | 9 |

| Web Table 2 Primary outcome (ipsilateral recurrent ischaemic stroke, post PET and before/without revascularisation) by category of carotid stenosis, and relative to revascularisation. | | | | |
| --- | --- | --- | --- | --- |
| Carotid stenosis | Total  n=212 | Primary outcome  n=16  (post PET,  Pre-Revascularisation or no revascularisation) | Primary outcome, occurring before vascularisation  n=6 | Primary outcome, no revascularisation performed  n=10 |
| <50% | 45 (21.2%) | 2 | 1 | 1 |
| 50-69% | 93 (43.9%) | 8 | 4 | 4 |
| 70% | 74 (34.9%) | 6 | 1 | 5 |

| Web Table 3 Carotid Stenosis Category, Method of measurement, Presence or absence of Carotid revascularisation. | | | | | |
| --- | --- | --- | --- | --- | --- |
| Carotid stenosis  (NASCET) | Total number (%) | Stenosis severity determined by CT Angiography | Stenosis severity determined by Carotid doppler Ultrasound or MR Angiography | Revascularisation performed  (CEA or CAS) | No revascularisation (CEA or CAS) performed |
| Total | 212 | 137 | 75 | 108 | 104 |
| <50% | 45 (21.2%) | 6 (13.3%) | 39 (86.7%) | 4 (8.9%) | 41 (39.4%) |
| 50-69% | 92 (43.4%) | 72 (78.3%) | 20 (21.7%) | 46 (50%) | 46 (44.2%) |
| ≥70% | 68 (32%) | 58 (85.3%) | 10 (14.7%) | 55 (80.9%) | 13 (12.5%) |
| Near occlusion | 7 (3.3%) | 1 (14.3%) | 6 (85.7%) | 3 (42.9%) | 4 (57.1%) |

| **Web Table 4 Imaging modality of carotid stenosis and revascularisation** | | |
| --- | --- | --- |
| Imaging Modality | No revascularisation  n=104 | Revascularisation  n=108 |
| Non CT  (carotid US or MR Angiography)  n=75 | 60 (80%) | 15 (20%) |
| CT Angiography  n=137 | 44 (32.1%) | 93 (67.9%) |

| **Web Table 5 Clinical Characteristics by Outcome (Ipsilateral Recurrent Ischaemic Stroke, post PET, pre or without revascularisation)** | | | |
| --- | --- | --- | --- |
|  | Outcome  n=16 | No Outcome  N=196 | p |
| Age (mean, SD) | 73 (SD 7.7) | 72 (SD 8.8) | 0.41 |
| Male | 10 (62.5%) | 144 (73.5%) | 0.34 |
| Hypertension | 14 (87.5%) | 166 (84.7.%) | 0.76 |
| Diabetes mellitus | 7 (43.8%) | 49 (25%) | 0.1 |
| Current smoking | 5 (31.3%) | 65 (33.2%) | 0.88 |
| Coronary artery disease | 1 (6.25%) | 43 (21.9%) | 0.14 |
| Antiplatelet at study recruitment | 16 (100%) | 158 (80.6%) | 0.05 |
| Statin at study recruitment | 14 (87.5%) | 156 (79.6%) | 0.45 |
| *Clinical event*  Retinal artery embolism  Transient ischaemic attack  Minor stroke (NIH <5)  Major stroke (NIH$\geq$5) | 0 (0%)  5(13.25%)  8(50%)  3(18.75%) | 12(6.1%)  88(44.9%)  84(42.9%)  12(6.1%) | 0.16 |
| *Carotid Artery stenosis*  *<50%*  *50-69%*  *≥70%*  *Near Occlusion* | 2 (12.5%)  8 (50%)  6 (37.5%)  0 (0%) | 43 (21.9%)  84 (42.9%)  62 (31.6%)  7 (100%) | 0.68 |
| SUV_max_, Median (IQR) | 3.2  (2.6-3.7) | 2.73  (2.3-3.2) | 0.03 |
| SCAIL, Median (IQR) | 3 (2-4) | 2 (2-3) | 0.03 |
| OCST, Median (IQR) | 17.1  (11.5-23.2) | 14.8  (8.7-32.4) | 0.8 |
| ESRS, Median (IQR) | 4 (3-4) | 3 (2-4) | 0.14 |
| **p=t-test for comparison of means; Chi^2^ for comparison of proportions; Kruskal-Wallis for comparison of medians* | | | |

**Additional results**

| **Web Table 6 Sensitivity analysis OCST assuming 25% ulcerated plaque** | | | |
| --- | --- | --- | --- |
|  | HR | 95% CI | P-value |
| OCST 25 % ulcerated, crude | 0.997 | 0.988-1.006 | 0.515 |
| OCST 25% ulcerated, adjusted Model 1 | 1.000 | 0.994-1.006 | 0.975 |

*Model 1: adjustment for statin and antiplatelet therapy*

| **Web Table 7 HR for ESRS when dichotomised into low-risk and high-risk categories** | | | |
| --- | --- | --- | --- |
|  | HR | 95% CI | p-value |
| ESRS dichotomised | 2.93 | 0.665-12.886 | 0.156 |
| ESRS dichotomised, Model 1 | 2.28 | 0.481-10.809 | 0.299 |

*Model 1: adjustment for statin and antiplatelet therapy*

| **Web table 8**  **Association of combinations of ESRS with stenosis alone and both SCAIL items, with recurrent ipsilateral ischaemic stroke (HR per 1 point increase)**  **(n=212, 16 recurrent ipsilateral ischaemic strokes)** | | | | | | |
| --- | --- | --- | --- | --- | --- | --- |
|  | **Crude** | | | **Adjusted for antiplatelet and statin** | | |
|  | **HR** | **CI** | **p** | **HR** | **CI** | **p** |
| ESRS+stenosis | 1.38 | 0.99-1.92 | 0.058 | 1.35 | 0.96-1.93 | 0.09 |
| ESRS+SCAIL | 1.6 | 1.14-2.25 | 0.007 | 1.57 | 1.10-2.25 | 0.01 |

| **Web table 9**  **Sensitivity and Specificity of Each Score and Combinations of Scores** | | | | |
| --- | --- | --- | --- | --- |
| **Score** | **Threshold** | **Sensitivity** | **Specificity** | **% correctly classified** |
| OCST | ≥Median value of 15.5 | 56.3% | 50.5% | 50.9% |
| ESRS | High risk score ≥3 | 87.5% | 31.1% | 35.4% |
| SCAIL | High risk score ≥3 | 75% | 52.5% | 53.3% |
| ESRS + SUV_max_ | High-risk category | 56.3% | 78.1% | 76.4% |
| ESRS + SCAIL | High-risk category | 68.8% | 69.4% | 69.3% |

| **Web Table 10**  **Comparison of Discrimination of each score and combination by comparison of**  **C-statistics** | | | | | | | | | |
| --- | --- | --- | --- | --- | --- | --- | --- | --- | --- |
|  | **SCAIL** | **ESRS** | **p** | **ESRS+ SUV_max_** | **p**  **(vs ESRS)** | **ESRS+**  **Stenosis** | **p**  **(vs ESRS)** | **ESRS+**  **SCAIL** | **p**  **(vs ESRS)** |
| c-statistic | 0.66 (0.51-0.8) | 0.61 (0.48-0.74) | 0.58 | 0.66 (0.52-0.8) | 0.2 | 0.62  (0.47-0.77) | 0.67 | 0.69 (0.55-0.82) | 0.22 |

**Web Figure 1 Kaplan Meier curve for recurrent ipsilateral ischaemic stroke (post-PET and pre or without revascularisation)**

1. **OCST (dichotomised at median value)**

1. **ESRS (dichotomised at <3 and ≥3)**

1. **SCAIL (dichotomised at <3 and ≥3)**

**Web Figure 2 Area under the receiver operating curve for comparison of c-statistics for SCAIL, OCST and ESRS.**

**
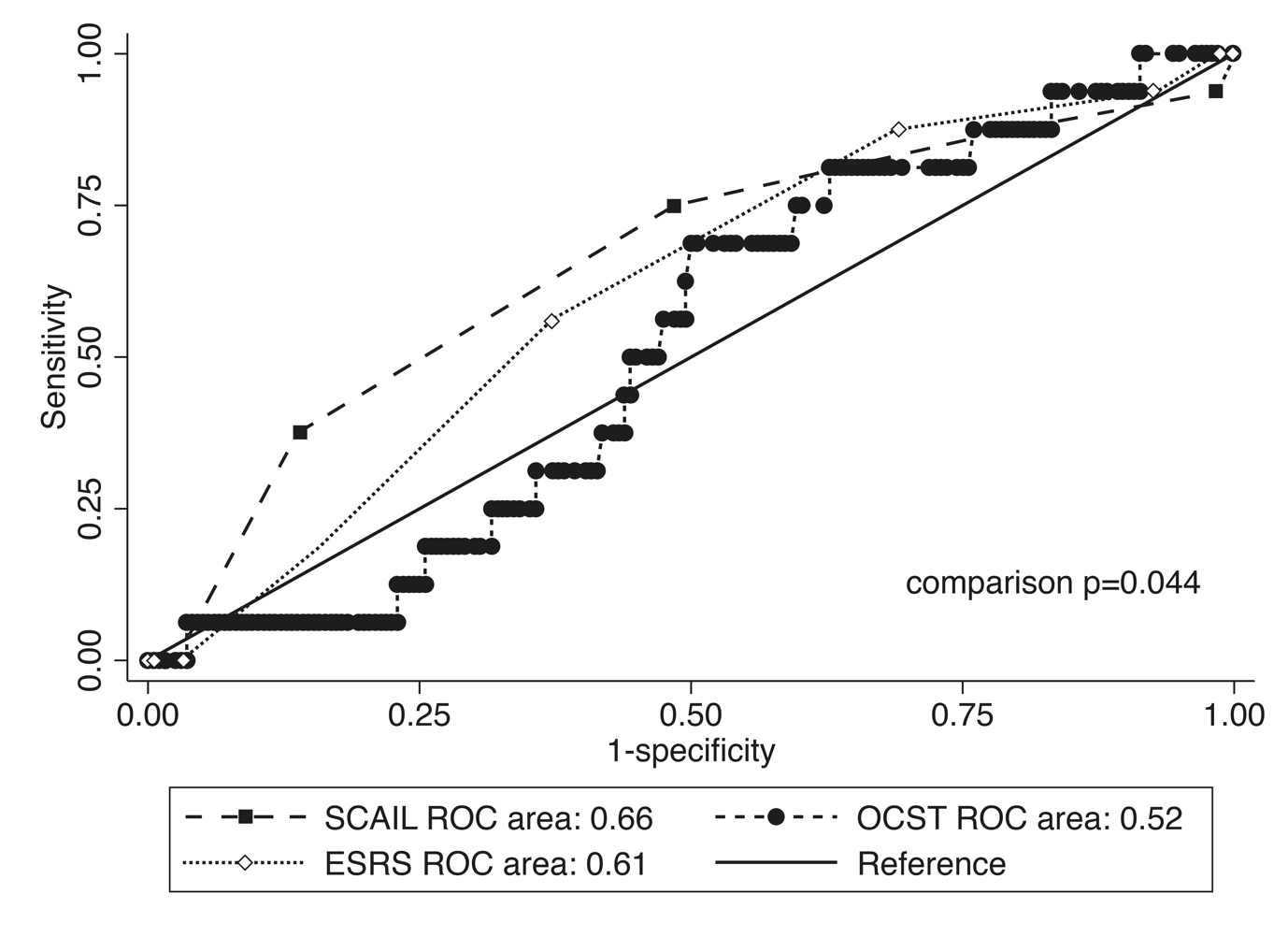
**

|  |  | Comparison (chi-squared) |
| --- | --- | --- |
| SCAIL c-statistic 0.66 | OCST c-statistic 0.52 | p=0.03 |
| SCAIL c-statistic 0.66 | ESRS c-statistic 0.62 | p=0.2 |

**Web Figure 3 Recurrent Ipsilateral Stroke rate per category created by combination of ESRS and SCAIL**

Categories defined by dichotomising ESRS and SCAIL as:

Low, ESRS<3+SCAIL <3;

Moderate, ESRS≥3+SCAIL<3 *or* ESRS<3 +SCAIL ≥3;

High, ESRS≥3+SCAIL ≥3.

**Web Figure 4 Net Reclassification Matrix**

**Web Figure 4 Net Reclassification Matrix**

A) movement in categories from ESRS categories low-ESRS <3 and high-ESRS ≥3 to ESRS and SUV_max_ combined categories of low (low-ESRS<3 and low-SUV_max_<3), moderate (low-ESRS<3 and high-SUV_max_≥3 *or* high-ESRS≥3 and low-SUV_max_<3) and high (high-ESRS≥3 and high-SUV_max_≥3)

B) movement in categories from ESRS categories low-ESRS<3 and high-ESRS ≥3 to ESRS and SCAIL combined categories of low (low-ESRS<3 and low-SCAIL<3), moderate (low-ESRS<3 and high-SCAIL≥3 or high-ESRS≥3 and low-SCAIL<3) and high (high-ESRS≥3 and high-SCAIL≥3)

**Additional references:**

1. McCabe JJ, Camps-Renom P, Giannotti N, et al. Carotid Plaque Inflammation Imaged by PET and Prediction of Recurrent Stroke at 5 Years. *Neurology* 2021; **97**(23): e2282-e91.

2. Camps-Renom P, McCabe J, Martí-Fàbregas J, et al. Association of Plaque Inflammation With Stroke Recurrence in Patients With Unproven Benefit From Carotid Revascularization. *Neurology* 2022; **99**(2): e109-e18.

3. Barnett HJ, Taylor DW, Eliasziw M, et al. Benefit of carotid endarterectomy in patients with symptomatic moderate or severe stenosis. North American Symptomatic Carotid Endarterectomy Trial Collaborators. *N Engl J Med* 1998; **339**(20): 1415-25.

4. Bucerius J, Hyafil F, Verberne HJ, et al. Position paper of the Cardiovascular Committee of the European Association of Nuclear Medicine (EANM) on PET imaging of atherosclerosis. *Eur J Nucl Med Mol Imaging* 2016; **43**(4): 780-92.

5. Boellaard R, O'Doherty MJ, Weber WA, et al. FDG PET and PET/CT: EANM procedure guidelines for tumour PET imaging: version 1.0. *Eur J Nucl Med Mol Imaging* 2010; **37**(1): 181-200.

6. Giannotti N, O'Connell MJ, Foley SJ, Kelly PJ, McNulty JP. Carotid atherosclerotic plaques standardised uptake values: software challenges and reproducibility. *EJNMMI Res* 2017; **7**(1): 39.

7. Weimar C, Diener HC, Alberts MJ, et al. The Essen stroke risk score predicts recurrent cardiovascular events: a validation within the REduction of Atherothrombosis for Continued Health (REACH) registry. *Stroke* 2009; **40**(2): 350-4.

8. Diener HC, Ringleb PA, Savi P. Clopidogrel for the secondary prevention of stroke. *Expert Opin Pharmacother* 2005; **6**(5): 755-64.
